# Identification and biochemical characterization of a novel *N*-acetylglucosamine kinase in *Saccharomyces cerevisiae*

**DOI:** 10.1038/s41598-022-21400-3

**Published:** 2022-10-10

**Authors:** Midori Umekawa, Ayano Nishikawa, Naoto Isono, Shuichi Karita

**Affiliations:** Graduate School of Bioresources, Mie University, Tsu, 514-8507 Japan

**Keywords:** Microbiology, Fungi

## Abstract

*N*-acetylglucosamine (GlcNAc) is a key component of glycans such as glycoprotein and the cell wall. GlcNAc kinase is an enzyme that transfers a phosphate onto GlcNAc to generate GlcNAc-6-phosphate, which can be a precursor for glycan synthesis. GlcNAc kinases have been found in a broad range of organisms, including pathogenic yeast, human and bacteria. However, this enzyme has never been discovered in *Saccharomyces cerevisiae*, a eukaryotic model. In this study, the first GlcNAc kinase from *S. cerevisiae* was identified and named Ngk1. The *K*_m_ values of Ngk1 for GlcNAc and glucose were 0.11 mM and 71 mM, respectively, suggesting that Ngk1 possesses a high affinity for GlcNAc, unlike hexokinases. Ngk1 showed the GlcNAc phosphorylation activity with various nucleoside triphosphates, namely ATP, CTP, GTP, ITP, and UTP, as phosphoryl donors. Ngk1 is phylogenetically distant from known enzymes, as the amino acid sequence identity with others is only about 20% or less. The physiological role of Ngk1 in *S. cerevisiae* is also discussed.

## Introduction

*N*-acetylglucosamine (GlcNAc), a ubiquitous carbohydrate in both prokaryotes and eukaryotes, is an essential component of glycans such as *N*-glycoproteins, GPI-anchors, and cell walls, including peptidoglycans of bacteria and chitins of yeasts^1–3^. For glycan biosynthesis, uridine diphosphate *N*-acetylglucosamine (UDP-GlcNAc) is an essential substrate in both prokaryotes and eukaryotes because the GlcNAc moiety of this nucleotide sugar is incorporated into glycans by glycosyltransferases^2,3^. Generally, UDP-GlcNAc is synthesized from fructose-6-phosphate (Fru-6-P), a glycolysis intermediate, which can be converted into glucosamine-6-P^3^. In eukaryotes, glucosamine-6-P is acetylated to be GlcNAc-6-P, followed by the isomerization to GlcNAc-1-P^3^*.* In prokaryotes, on the other hand, glucosamine-6-P is primarily isomerized into glucosamine-1-P, and then acetylated to be GlcNAc-1-P^3,4^. In the final step, GlcNAc-1-P is converted into UDP-GlcNAc by uridylation in both prokaryotes and eukaryotes^3,4^.

GlcNAc kinase (EC 2.7.1.59) is a GlcNAc-metabolizing enzyme discovered in broad species of organisms, including bacteria, pathogenic yeast, plants, and animals^3–8^. This enzyme transfers the gamma-phosphoryl group of an ATP onto the hydroxyl group at the C-6 of GlcNAc to generate a GlcNAc-6-P. GlcNAc kinase is structurally related to hexokinase (EC 2.7.1.1), which is another type of sugar kinase that acts preferentially on glucose (Glc) to generate Glc-6-P at the first step of glycolysis, as these enzymes commonly have an ATPase domain^9,10^. In mammals, GlcNAc kinase is known as an enzyme that provides a salvage pathway of UDP-GlcNAc biosynthesis since this enzyme produces GlcNAc-6-P, which can be a precursor of UDP-GlcNAc^11^. In contrast, the role of NagK, the GlcNAc kinase of *Escherichia coli*, in GlcNAc utilization for the biosynthesis of Fru-6-P has been studied since there is a reversed pathway to convert GlcNAc-6-P into Fru-6-P for the energy source in glycolysis^4^. Similarly, *Ca*Nag5, the GlcNAc kinase of pathogenic yeast *Candida albicans*, was also identified and reported to be involved in GlcNAc utilization to generate Fru-6-P as an energy source^3,5,6^. Like bacteria, *C. albicans* can grow on GlcNAc as a carbon source. Indeed, there is a pathway to convert GlcNAc into Fru-6-P in *C. albicans*, because the gene cluster of GlcNAc-metabolizing enzymes, consisting of GlcNAc kinase (*CaNAG5*) as well as GlcNAc-6-P deacetylase (*CaNAG2*) and glucosamine-6-P deaminase (*CaNAG1*), was identified from the sequence similarity with these enzymes of *E. coli*^4^. It was shown that disruption of these genes retarded the growth of *C. albicans* on GlcNAc as a carbon source.

Unlike *C. albicans*, budding yeast *Saccharomyces cerevisiae* does not grow on GlcNAc as a carbon source^3,5,6^. Moreover, any gene similar to either of *CaNAG5, CaNAG2* or *CaNAG1* is not conserved in the whole genome of *S. cerevisiae*^6^*.* Therefore, it has been considered that *S. cerevisiae* does not possess these GlcNAc-metabolizing enzymes*.* In *S. cerevisiae*, the presence of three hexokinases, Hxk1, Hxk2, and Glk1, has been known since the 1980s^12^. It has been believed for several decades that *S. cerevisiae* does not possess another hexokinase other than Hxk1, Hxk2, and Glk1. However, we have noticed and focused on the presence of two other hexokinase-like genes (but whose functions were unknown): *EMI2* and *YLR446W*. In our previous work, the Emi2 protein was identified as a fourth new hexokinase of *S. cerevisiae* in addition to the previously known ones: Hxk1, Hxk2, and Glk1^13^. In this study, we focused on another hexokinase-like gene, *YLR446W* (systematic name), which has no standard gene name, to examine whether it is an additional new hexokinase in this model organism. Unexpectedly, we found that *YLR446W* encodes a novel GlcNAc kinase that shows low sequence similarity to known GlcNAc kinases from other organisms.

## Results

### The YLR446W protein is a kinase with low glucose phosphorylation activity

The *YLR446W* gene is a function-unknown gene that encodes a protein of 433 amino acids and has a hexokinase-like ATPase domain. A homology search did not find any function-known enzyme having high similarity with the protein encoded by the *YLR446W* gene (YLR446Wp). However, YLR446Wp has an approximately 20% sequence identity with Hxk1, Hxk2, Glk1, and Emi2 of *S. cerevisiae* (Supplementary Fig. S1). In addition, this gene product was predicted to be about 50 kDa of a cytosolic protein that has no signal peptide sequence, as is the case with Hxk1, Hxk2, Glk1, and Emi2^9,13^. Therefore, we started to examine whether this gene encodes a hexokinase. The recombinant YLR446Wp was expressed in *E. coli* cells and purified for use in an enzymatic assay (data not shown).

**Figure 1:**
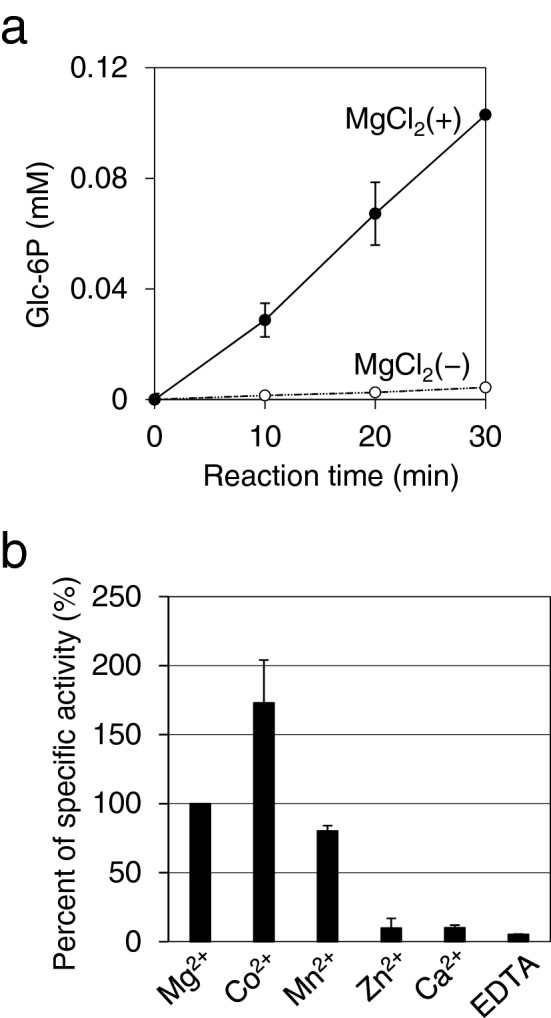
Assay of Glc phosphorylation activity of YLR446Wp. (**a**) The enzyme activity of YLR446Wp for glucose phosphorylation was monitored using a glucose-6-phosphate dehydrogenase-coupled assay. The reaction of glucose phosphorylation with Ngk1 was assessed in the presence (+) or absence (−) of MgCl_2_. (**b**) The specific activity for glucose phosphorylation in the presence of either MgCl_2_, CoCl_2_, MnCl_2_, ZnCl_2_, CaCl_2_, or EDTA was compared. The percentage of the specific activity in the presence of MgCl_2_ was set as 100%. Error bars, the standard deviation of two independent experiments.

Typically, ATP can be a phosphoryl donor for hexokinase by forming a complex with divalent cations, such as Mg^2+^. It was found that YLR446Wp showed low glucose phosphorylation activity (0.010 U/mg) in the presence of ATP and Mg^2+^ (Fig. 1a). The relative activity levels of glucose phosphorylation in the presence of ATP and either of Mg^2+^, Co^2+^, or Mn^2+^ were 100%, 170% and 80%, respectively (Fig. 1b), suggesting that Mg^2+^ can be substituted by Co^2+^ or Mn^2+^. On the other hand, the enzyme activity was hardly detected in the presence of Ca^2+^ or Zn^2+^. In the absence of divalent cations, the enzyme activity was not observed in the presence of ATP (Fig. 1a and b). These results showed that YLR446Wp is a kinase that acts on Glc, with a requirement for divalent cations, similar to the case with known hexokinases^13^. It was also found that the glucokinase activity of this enzyme in the presence of 10 mM Glc was lower than those of the other four hexokinases, whose values were 0.044–180 mU for Hxk1, Hxk2, Glk1, and Emi2 under our assay conditions. Although the highest activity of YLR446Wp was observed in the presence of Co^2+^, only a trace amount of intracellular Co^2+^ was detected^14^. On the other hand, Mg^2+^ is abundant in yeast cells and is known as a major co-factor for ATP-dependent kinases^15^. Therefore, we decided to carry out the enzyme assay in the presence of Mg^2+^ in the subsequent experiments.

### The YLR446W protein is a GlcNAc kinase, named Ngk1

**Figure 2:**
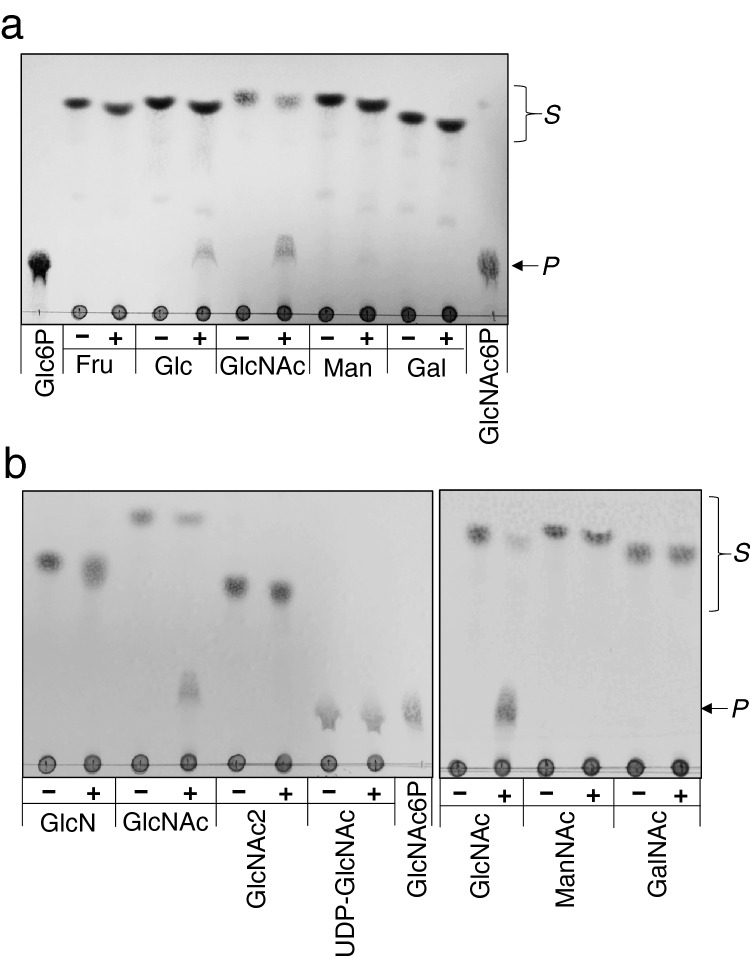
TLC analyses of sugar substrate specificity of YLR446Wp. The substrate specificity of the enzyme for monosaccharides (**a**) and amino sugars (**b**) was monitored by TLC. A reaction containing ATP, MgCl_2_, and each of the following sugars: GlcNAc, Glc, fructose (Fru), mannose (Man), galactose (Gal), glucosamine (GlcN), *N’N’*-diacetylchitobiose (GlcNAc2), UDP-GlcNAc, *N*-acetylmannosamine (ManNAc), or *N*-acetylgalactosamine (GalNAc) was carried out for 30 min with (+) or without (−) enzyme. Glc-6-P or GlcNAc-6-P (15 nmol each) was applied to the TLC plate as the standard compound. *S*, sugar substrates; *P*, products.

Most of the known hexokinases, including Hxk1, Hxk2, and Emi2, can act on not only glucose but also mannose, fructose and glucosamine^13,16^. We examined whether YLR446Wp can phosphorylate monosaccharides other than glucose by monitoring the reaction product by TLC. The phosphorylation product was hardly detected when either fructose, mannose, or galactose was used as a sugar substrate, suggesting that this enzyme does not recognize these monosaccharides as a substrate. In contrast, this enzyme demonstrates the phosphorylation activity on GlcNAc, since the phosphorylation product was generated with a decrease in the sugar substrate after a 30 min reaction (Fig. 2a). The phosphorylation of other amino sugars, glucosamine, *N*’*N*’-diacetylchitobiose, UDP-GlcNAc, *N*-acetylmannosamine and *N*-acetylgalactosamine, was also examined to find that this enzyme does not show detectable activity on these amino sugars other than GlcNAc (Fig. 2b). In contrast, none of the other yeast hexokinases, Hxk1, Hxk2, Glk1, and Emi2, showed the detectable activity on GlcNAc (Supplementary Fig. S2). On the other hand, all these hexokinases clearly exhibited the activity for Glc.

**Figure 3:**
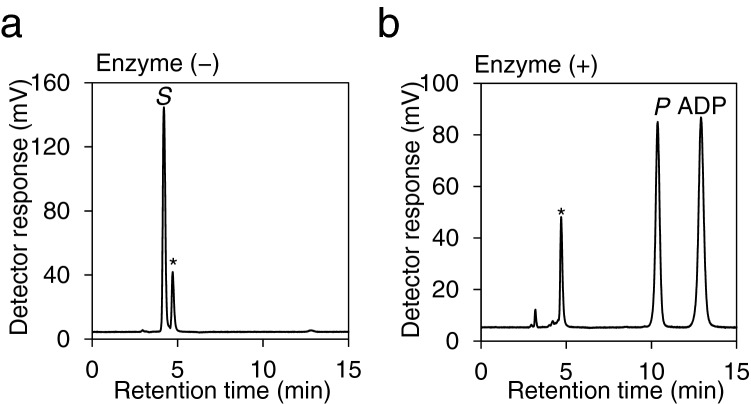
HPLC analysis of GlcNAc phosphorylation products. The reaction mixture in the absence (**a**) or presence (**b**) of the enzyme, comprising 10 mM GlcNAc, 10 mM ATP, and 10 mM MgCl_2_, was carried out in 50 mM Tris–HCl (pH 8.0) at 30 °C for 3 h and analyzed by HPLC. The retention time of each peak detected at UV = 205 nm was matched to that of the standard compounds: *S*, GlcNAc (4.2 min); *P*, GlcNAc-6-P (10.4 min); ADP (12.4 min); *, Tris (4.7 min).

To confirm that YLR446Wp possesses GlcNAc kinase activity, the reaction product was analyzed by HPLC. After 3 h of reaction with the enzyme, the peak of GlcNAc (*S*: RT = 4.19 min) was diminished and largely replaced by the product (*P*: RT = 10.39 min) (Fig. 3a and b). The retention time of the reaction product (*P*) was essentially matched to that of GlcNAc-6-P. It is noted that GlcNAc-1-P, another GlcNAc phosphate, was eluted at 9.87 min under the same condition (data not shown). In addition, ESI–MS spectrometry showed that the molecular mass of the reaction product (*P*: RT = 10.39 min) was matched to the calculated mass of GlcNAc-6-P [*m/z* [M − H]^−^ 300]. These results suggest that YLR446Wp possesses GlcNAc kinase activity to generate GlcNAc-6-P, unlike the other known hexokinases in *S. cerevisiae*.

**Figure 4:**
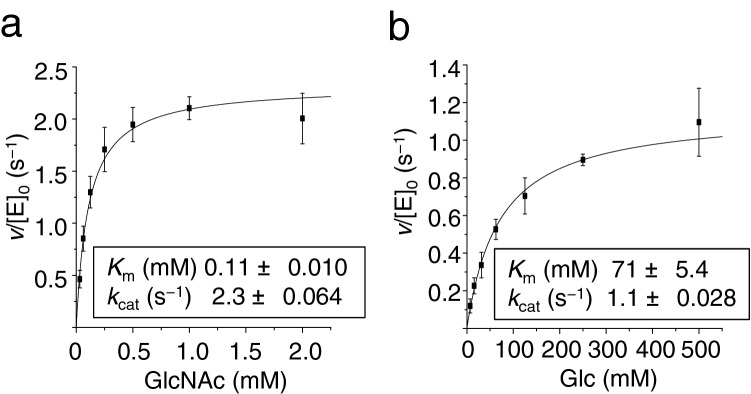
*YLR446W* encodes a GlcNAc kinase named Ngk1. Kinetic analysis of Ngk1 kinase activity of GlcNAc (**a**) and Glc (**b**) was carried out using a pyruvate kinase and lactate dehydrogenase coupled assay.

The kinetic parameters for GlcNAc and Glc were compared by monitoring the generation of ADP by the ATP-dependent sugar phosphorylation, through the use of the pyruvate kinase and lactate dehydrogenase coupled assay. Before the assay, we confirmed that the maximum enzyme activity was obtained around pH 8.0 using Tris–HCl buffer. The *K*_m_ values for GlcNAc and Glc were determined as 0.11 mM and 71 mM, respectively, indicating that the affinity of this kinase for GlcNAc is about 500 times higher than that of Glc (Fig. 4a and b). However, the turnover rates for these sugars were not significantly different: the *k*_cat_ values for GlcNAc and Glc were 2.3 s^−1^ and 1.1 s^−1^, respectively. The *K*_m_ and *k*_cat_ values of Ngk1 for ATP, with the use of GlcNAc as the sugar substrate, were determined as 1.2 mM and 3.4 s^−1^, respectively (data not shown). The *K*_m_ values of the other four hexokinases of *S. cerevisiae* for glucose phosphorylation were reported to be less than 0.20 mM (0.011–0.20 mM), whereas the *k*_cat_ values of these enzymes crossed a broad range (0.042–63 s^−1^)^13,16^. From these results, we have concluded that *YLR446W* does not encode a typical hexokinase but encodes a novel sugar kinase that is specific to GlcNAc and that has never been identified in *S. cerevisiae*. Accordingly, we named this gene *NGK1*, for *N*-acetylglucosamine kinase 1.

### Ngk1 is a GlcNAc kinase reacted with broad species of nucleoside triphosphate as a phosphoryl donor

**Figure 5:**
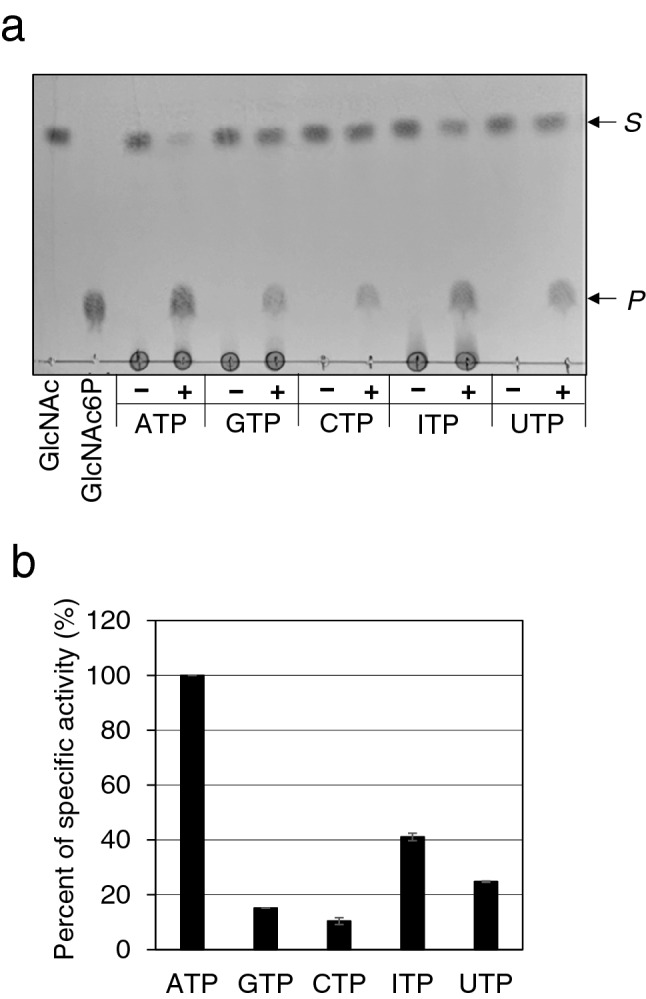
Analysis of the reactivity of Ngk1 to different nucleoside triphosphates. The Ngk1 activity of GlcNAc phosphorylation using either ATP, GTP, CTP, ITP, or UTP as a phosphoryl donor was determined. (**a**) TLC analysis of the reaction (40 min) with (+) or without (−) enzyme. *S*, sugar substrates; *P*, products. (**b**) The specific activities were determined by measuring the GlcNAc-6-P concentration using HPLC after the reaction (20 min) with different nucleoside triphosphates. The specific activity of Ngk1 against ATP was set as 100%. Error bars, the standard deviation of two independent experiments.

There is little knowledge about the reactiveness of GlcNAc kinases on nucleoside triphosphates other than ATP. It was reported that yeast cells contain non-ATP nucleoside triphosphates, CTP, GTP, ITP, and UTP^17^. We thus examined the Ngk1 activity with each of these nucleoside triphosphates as a phosphoryl donor. The phosphorylation product was observed by using any nucleoside triphosphates, including ATP, GTP, CTP, ITP, and UTP, after the reaction with Ngk1 (Fig. 5a). The relative activities against GTP, CTP, ITP, and UTP were 15%, 10%, 41% and 25%, respectively, compared to ATP (Fig. 5b). The retention time of each product in HPLC was consistent with that of the GlcNAc-6-P (data not shown). These data suggest that Ngk1 can utilize broad species of nucleoside triphosphates to generate GlcNAc-6-P*.*

### Ngk1 is a novel GlcNAc kinase having low similarity with other known GlcNAc kinases

It was difficult to align the Ngk1 sequence with GlcNAc kinases of the known primary structure (e.g., human and bacterial GlcNAc kinases) because of their low sequence identities (less than 15%). Since it is presumed that GlcNAc kinase would be functionally and structurally similar to hexokinases, we searched for potential amino acid residues involved in the reaction by alignment with the known hexokinases of *S. cerevisiae* (Supplementary Fig. S1). Among them, the structure–function relationships of Hxk2 have been well studied^18^. The multiple alignment indicated that Asp-196 and Lys-152 of Ngk1 may be the putative catalytic and ATP-binding residues, respectively. The replacement of either Asp-196 or Lys-152 by Ala (D196A or K152A) severely diminished the enzyme activity (Supplementary Fig. S3). D196E still accounted for a portion of the enzyme activity, whereas D196A and D196N did not show detectable activity after overnight reaction. These results suggest that the crucial residues for enzyme catalysis may be conserved in Ngk1, while the overall identity of Ngk1 with other hexokinases of *S. cerevisiae* is only about 20%.

**Figure 6:**
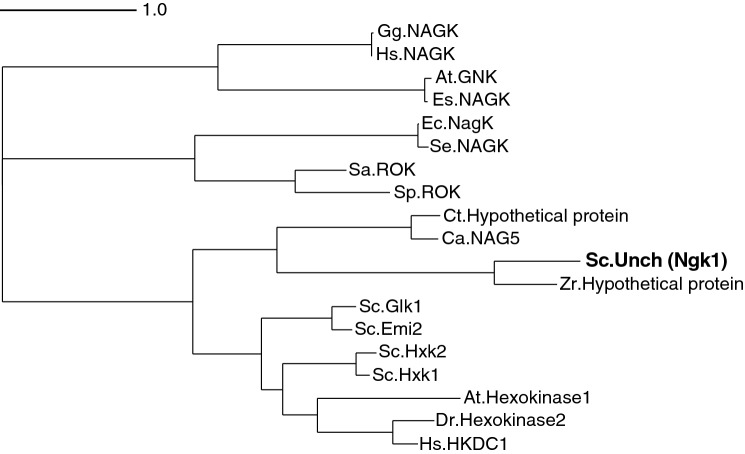
Phylogenic analysis of Ngk1 with GlcNAc kinases and hexokinases. Phylogenic tree of identified or putative GlcNAc kinases and hexokinases. A phylogenetic tree was created using Clustal Omega^19^ and visualized with Dendroscope 3^20^. Ngk1 and hexokinases of *S. cerevisiae*: Sc.Unch (Ngk1) (AAT92658.1), Sc.Hxk1 (NP_116711.3), Sc.Hxk2 (NP_011261.1), Sc.Glk1 (NP_009890.1), Sc.Emi2 (NP_010804.3). GlcNAc kinases of *C. albicans* (Ca.NAG5, BAB43816.1), human (Hs.NAGK, EAW99780.1), *E. coli* (Ec.NagK, NP_415637), *Salmonella enterica* (Se.NagK, EDN6582917.1), *Arabidopsis thaliana* (At.GNK, NP_564358.1), *Eutrema salsugineum* (Es.NAGK, XP_006415457.1), and *Gorilla gorilla gorilla* (Gg.NAGK, XP_018876635.1). *N*-acetylmannosamine kinases of *Staphylococcus aureus* (Sa.ROK, WP_000291445.1) and *Staphylococcus pseudintermedius* (Sp.ROK, WP_214533982.1). Hexokinases of higher eukaryotes, human (Hs.HKDC1, NP_079406.4), *Arabidopsis thaliana* (At.Hexokinase1, NP_194642.1), and *Danio rerio* (Dr.Hexokinase-2, NP_998231.1). Hypothetical proteins of *Candida tropicalis* (Ct.Hypothetical protein, XP_002549429.1) and *Zygosaccharomyces rouxii* (Zr.Hypothetical protein, GAV52624.1).

The phylogenic analysis shows that Ngk1 is distant from other known GlcNAc kinases as well as hexokinases (Fig. 6). Among the known enzymes, the GlcNAc kinase from *C. albicans* (Ca.NAG5) was the closest to Ngk1, although the amino acid sequence identity between them is only 22%. Other known GlcNAc kinases from both higher eukaryotes (Hs.NAGK and At.GNK, from human and *Arabidopsis thaliana*, respectively) and bacteria^4^ (Ec.NagK from *E. coli*) were obviously far from *S. cerevisiae* Ngk1 since their sequence identities with Ngk1 were less than 15%. It is noted that genes similar to *Ngk1* are conserved as hypothetical proteins in yeasts of the *Saccharomycetaceae* family, such as *Zygosaccharomyces rouxii* (Zr.Hypothetical protein, 41% identical to Ngk1), although none of them has been characterized.

## Discussion

In this study, we identified the first GlcNAc kinase from *S. cerevisiae*, a eukaryotic model organism. So far, GlcNAc kinases have been identified from a broad range of organisms, including bacteria and animals. However, it has been thought that *S. cerevisiae* has no GlcNAc kinase, since there is no gene similar to the known GlcNAc kinases in the whole genome. Indeed, we could not predict that the function-unknown, unnamed gene *YLR446W* of *S. cerevisiae* is a gene of GlcNAc kinase from the amino acid sequence because the sequence similarity with the known GlcNAc kinases is about 20% or less. Here, we determined the biochemical property of YLR446Wp and found that this gene does code a GlcNAc kinase, and thereby named it Ngk1.

Our enzymatic analysis showed that Ngk1 possesses kinase activity for both GlcNAc and Glc, although the *K*_m_ values for GlcNAc and Glc were 0.11 mM and 71 mM, respectively (Fig. 4a,b). Considering that the intracellular glucose concentration in *S. cerevisiae* cells is estimated to be ~ 1.5 mM^21^, Ngk1 may not be involved in glucose phosphorylation under the physiological conditions. There are a few reports on biochemical analyses of GlcNAc kinases from other organisms; the *K*_m_ values of GlcNAc kinases from *C. albicans* and human were reported to be 0.38 mM and 0.45 mM, respectively^6,8^. Therefore, the affinity of Ngk1 for GlcNAc is higher than that for other GlcNAc kinases. Ngk1 did not show the kinase activity for any sugars, except for GlcNAc and Glc (Fig. 2a,b). Human GlcNAc kinase acts on ManNAc as well as Glc, though to a lesser extent than GlcNAc^8^. On the other hand, *C. albicans* Nag5 has a partial activity for Glc and mannose but not for ManNAc^6^. Ngk1 showed the activity with any nucleotide sugars, e.g. GTP, CTP, ITP, and UTP (Fig. 5a), suggesting that Ngk1 may utilize a broad range of nucleoside triphosphates as a phosphoryl donors. Although there are few reports on the reactiveness of sugar kinases for various nucleoside triphosphates, it was reported that CaNag5 did not show the kinase activity with these nucleotide sugars except for ATP and CTP^5^. Taken together, these results suggest that the enzymatic property of GlcNAc kinases is different from those of its species. GlcNAc kinases from different species are distant phylogenetically, whereas hexokinases from different species are relatively near (Fig. 6). Hence, GlcNAc kinases from different species may be derived from the distant origins and evolved uniquely. Notably, genes similar to *Ngk1* are conserved across different genera of fungi, e.g., *Z. rouxii*, as hypothetical or functional unknown proteins. Ngk1 of *S. cerevisiae* may be a clue toward the discovery of a new cluster of GlcNAc kinases.

The physiological significance of GlcNAc kinases is obscure, although this enzyme has been found in a broad range of organisms, from bacteria to human. In *C. albicans*, it was reported that disruption of Nag5 caused an attenuation of its virulence in mice as well as a reduction in the growth on GlcNAc medium^6^. It is supposed that GlcNAc kinases of mammals provide a salvage pathway for UDP-GlcNAc synthesis by phosphorylating intracellular GlcNAc that may be derived from lysosomes^11^, but little experimental evidence has been reported. In *E. coli*, NagK is known to play a role in utilizing intracellular GlcNAc derived from cell wall murein degradation, since extracellular GlcNAc is phosphorylated during its transport into the cells^4^. At least, Ngk1 of *S. cerevisiae* may not contribute to GlcNAc metabolism for glycolysis, as this organism does not grow by utilizing extracellular GlcNAc as a carbon source^5^. One possibility is that Ngk1 may contribute to UDP-GlcNAc biosynthesis by phosphorylating GlcNAc in the cell. However, it is still uncertain that intracellular free GlcNAc can exist either from outside or inside of the cells because pathways for supplying intracellular GlcNAc have not been identified in this organism. Presumably, Ngk1 may be dispensable for vegetative growing cells in the presence of enough glucose because UDP-GlcNAc is synthesized from Fru-6-P via the known pathway. In fact, yeast cells lacking Ngk1 grew normally in the presence of enough glucose (data not shown). Given that a genome-wide transcriptome suggested that the transcript of this gene is upregulated during sporulation and unfolded protein response^22,23^, Ngk1 may play a role under a certain condition as an alternative supply pathway GlcNAc-6-P as a precursor of UDP-GlcNAc. Analyses to elucidate the physiological role of Ngk1 in *S. cerevisiae* are under way.

## Methods

### Preparation of the recombinant Ngk1 protein and other hexokinases

A full-length DNA fragment of the ORF region of *YLR446W* was amplified by PCR using the genomic DNA of the *S. cerevisiae* BY4742 strain (Invitrogen, Waltham, MA, USA) as a template and oligonucleotide primers (forward: 5’-ATTTATCATATGACAATTGAAAGCACTCTAGCTCGGG -3’; reverse: 5’-ATTTATCTCGAGTTATTGAACTTGGTTGTCTGATTTGTTCAAGTAGGTG-3’). The amplified DNA fragment was digested with *Nde* I and *Xho* I and ligated into the corresponding sites of a pCold II vector (Takara Bio, Shiga, Japan) to be fused with a His_6_-tag at the N-terminus. The nucleotide sequence of the resultant plasmid (pColdII-His_6_-Ngk1) was confirmed by DNA sequencing. Then, *E. coli* BL21 (DE3) was transformed with pColdII-His_6_-Ngk1, and the recombinant protein was expressed according to the manufacturer’s protocol. The cells were harvested and sonicated on ice in 50 mM Tris–HCl buffer (pH 8.0) containing 150 mM NaCl, 1 mM phenylmethylsulfonyl fluoride, and 1 mM dithiothreitol. The cell-free extract was applied to a HisTrap™ HP column (GE Healthcare, Chicago, IL, USA), and the protein was purified according to the manufacturer’s protocol. The protein concentration was measured using Protein Assay CBB solution (Nacalai Tesque, Kyoto, Japan). Recombinant proteins of Hxk1, Hxk2, Glk1 and Emi2 were prepared as reported previously^13^.

The site-directed mutagenesis of Ngk1 was carried out by PCR using pColdII-His_6_-Ngk1 as a template and the oligonucleotide primers listed in supplementary Table S1, according to the procedure described for the QuikChange site-directed mutagenesis kit (Stratagene, Germany). Each plasmid's mutation was confirmed by DNA sequencing. Using the resultant plasmids, each mutant enzyme of Ngk1 was expressed in *E. coli* BL21 (DE3) and used for enzyme assay after purification, as described above for the wild-type enzyme.

### Measurement of glucokinase activity

Measurement of the glucokinase activity of Ngk1 was carried out by the coupling reaction of the glucose-6-phosphate dehydrogenase (Oriental Yeast, Tokyo, Japan) with the conversion of NAD^+^ into NADH^13,24^. The assay mixture comprised 10 mM glucose, 5 mM ATP, 5 mM MgCl_2_, 2 mM NAD^+^, 50 mM Tris–HCl (pH 8.0), 1.6 U/mL glucose-6-phosphate dehydrogenase, and an appropriate amount of the enzyme. To examine the effect of divalent cations, 5 mM MgCl_2_ was replaced by either of the same concentration of CoCl_2_, MnCl_2_, ZnCl_2_, CaCl_2_ or by 1 mM EDTA. The reaction at 30 °C was started by adding the enzyme and followed by monitoring the increase in absorbance at 340 nm for NADH production. One unit (U) is defined as the amount of the enzyme that catalyzes the phosphorylation of 1 μmol of Glc per minute at 30 °C.

### Substrate specificity assay

To examine the substrate specificity of the enzyme for different sugars, a reaction comprising 10 mM sugar (either of GlcNAc, glucose, mannose, fructose, galactose, glucosamine, *N*-acetylmannosamine, UDP-GlcNAc or *N*’*N*’-diacetylchitobiose), 10 mM ATP, and 10 mM MgCl_2_ was carried out at 30 °C in 50 mM Tris–HCl (pH 8.0) with or without enzyme for the indicated time. For enzyme assays with different nucleoside triphosphates as phosphoryl donors, ATP was replaced by either nucleoside triphosphates (GTP, CTP, ITP, or UTP) using GlcNAc as a sugar substrate. The reaction was stopped by heating at 100 °C for 3 min. The reaction products were analyzed by TLC and HPLC. One unit (U) is defined as the amount of the enzyme that catalyzes the phosphorylation of 1 μmol of GlcNAc per minute at 30 °C.

### TLC analysis

A sample (3 µL) was spotted on a TLC silica gel 60 F_254_ plate (Merck, Darmstadt, Germany). The plate was developed in 1-butanol/acetic acid/water (8/3/2), dried, and soaked in a color reagent (2% diphenylamine, 2% aniline, and 85% phosphoric acid) before baking at 100 °C for 10 min. Commercial Glc-6-P, GlcNAc-6-P, and GlcNAc were used as standards.

### HPLC and MS analyses

The reaction products were analyzed by HPLC using a COSMOSIL HILIC column (Nacalai Tesque, Japan) and a UV detector at 205 nm. A mixture of sodium phosphate buffer (10 mM, pH 7.0) and acetonitrile (40:60, v/v) was used as the mobile phase at a flow rate of 1.0 mL/min, and the column temperature was set at 30 °C. Commercial GlcNAc, GlcNAc-6-P, GlcNAc-1-P, ADP, and Tris were used as standards. GlcNAc-6-P was quantified from the integrated peak areas. ESI–MS was also carried out to determine the mass of the molecule presumed to be GlcNAc-6-P in the reaction products using an LCMS-2010EV mass spectrometer (Shimadzu, Kyoto, Japan).

### Kinetic assay

To determine the kinetic parameters, the amount of ADP formed by sugar phosphorylation was measured using a pyruvate kinase and lactate dehydrogenase coupled assay, as established previously^13,24^. The assay mixture comprised six to seven different concentrations of GlcNAc (0.031–2.0 mM) or glucose (7.3–500 mM) as a sugar substrate, 5 mM ATP, 5 mM MgCl_2_, 5 mM phosphoenolpyruvate, 0.3 mM NADH, 50 mM Tris–HCl (pH 8.0), 18 U/mL pyruvate kinase (Oriental Yeast, Japan), 20 U/mL lactate dehydrogenase (Oriental Yeast, Japan) and an appropriate amount of Ngk1. The reaction at 30 °C was started by adding Ngk1 and followed by monitoring the decrease of NADH in absorbance at 340 nm. The *K*_m_ and *k*_cat_ values were determined by nonlinear regression analysis with Origin software (OriginLab, Northampton, MA, USA).

## Supplementary Information

Supplementary Information.

## Data Availability

The datasets generated and/or analyzed in this study are available from the corresponding author upon reasonable request.

## Abbreviations

Fru: Fructose
Glc: Glucose
GlcNAc: *N*-acetylglucosamine
1-P: 1-Phosphate
6-P: 6-Phosphate
UDP-GlcNAc: Uridine diphosphate *N*-acetylglucosamine
