# Supplementary material for: Identification and biochemical characterization of a novel N-acetylglucosamine kinase in Saccharomyces cerevisiae
Source: Sci Rep. 2022 Oct 10;12:16991. doi: 10.1038/s41598-022-21400-3 (PMC9550789; doi:10.1038/s41598-022-21400-3)
Supplement: Supplementary file 1 — Supplementary Information. [file 41598_2022_21400_MOESM1_ESM.pdf]

Supplementary Information file

The title of the manuscript:
Identification and biochemical characterization of a novel *N*-acetylglucosamine kinase in *Saccharomyces cerevisiae*

Author list:
Midori Umekawa*, Ayano Nishikawa, Naoto Isono, Shuichi Karita

Graduate School of Bioresources, Mie University, Tsu 514-8507, Japan
*e-mail: midoriumekawa@bio.mie-u.ac.jp

Supplementary Table S1. Primers used for site-directed mutagenesis of Ngk1

Mutants and primer sequences
K152A
forward: 5'- GTTGCAATGGGAgcAGGATTTGTGATG-3'
reverse: 5'- CATCACAAATCCTgcTCCCATTGCAAC-3'
D196A
forward: 5'-GTGTGTCATGTAATAAACGcTGCAATTGCAGTTTCACTAAC-3'
reverse: 5'-GTTAGTGAAACTGCAATTGCAgCGTTTATTACATGACACAC-3'
D196N
forward: 5'- GTGTGTCATGTAATAAACaATGCAATTGCAG-3'
reverse: 5'- CTGCAATTGCATtGTTTATTACATGACACAC-3'
D196E
forward: 5'- GTGTGTCATGTAATAAACGAgGCAATTGCAG-3'
reverse: 5'- CTGCAATTGCcTCGTTTATTACATGACACAC-3'
E290A
forward: 5'- GAAATGCCACTAGcGTGCGTTACTTCTG-3'
reverse: 5'- CAGAAGTAACGCACgCTAGTGGCATTTC-3'

Supplementary Fig.S1

```
Sc.Unch    1 --------------------MTIESTLARELESLILPADSIVNVVDQFQEELLSRLQ---  37
Sc.Hxk1    1 MVHLGPKKPQARKGSMADVPKELMDEIHQLEDMFTVDSETLRKVVKHFIDELNKGLTKK-  59
Sc.Hxk2    1 MVHLGPKKPQARKGSMADVPKELMQQIENFEKIFTVPTETLQAVTKHFISELEKGLSKK-  59
Sc.Emi2    1 MSF---------ENLHKVNAEALEDAVVEICSSLQVDAAKLDELTAYFIECMEKGLNNTS  51
Sc.Glk1    1 MSF---------DDLHKATERAVIQAVDHICDDFEVTPEKLDELTAYFIEQMEKGLAPPK  51

Sc.Unch   38 -------TNTISMLPQCLVPDKRSRWNPEDKILTIDFGGTRLKFAIISLPQI--------  82
Sc.Hxk1   60 -------GGNIPMIPGWVMEFP--TGKESGNYLAIDLGGTNLRVVLVKLSGNHTFDTTQS 110
Sc.Hxk2   60 -------GGNIPMIPGWVMDFP--TGKESGDFLAIDLGGTNLRVVLVKLGGDRTFDTTQS 110
Sc.Emi2   52 VGEEKTVDKGLPMIPTYVTSLP--NGTERGVLLAADLGGTHFRVCSVTLNGDGTFDMQQL 109
Sc.Glk1   52 EGHTLASDKGLPMIPAFVTGSP--NGTERGVLLAADLGGTNFRICSVNLHGDHTFSMEQM 109

Sc.Unch   83 VIEYNDAFELTYNIVDSNFFNQIIYTICTRLAANGYIKKK------NESSEASKFFVSVT 136
Sc.Hxk1  111 KYKLPHDMRTTK--HQEELWSFIADSL------KDFMVEQELLNT------KDTLPLGFT 156
Sc.Hxk2  111 KYRLPDAMRTTQ--NPDELWEFIADSL------KAFIDEQFPQGI------SEPIPLGFT 156
Sc.Emi2  110 KSKIPEEYLNDKDVTSEELFSYLGRRT------RAFVRKHHPELLKSTGENIKPLKMGFT 163
Sc.Glk1  110 KSKIPDDLLDDENVTSDDLFGFLARRT------LAFMKKYHPDELA-KGKDAKPMKLGFT 162
                                 ▽
Sc.Unch  137 FSFPLNP----EGEVVAMGKGFVMTDTLQGSTVKQLIQSSFHRIISENIEEFFCTMNVCH 192
Sc.Hxk1  157 FSYPASQNKINEGILQRWTKGFDIPNVEGHDV-VPLLQNEISKR-------ELP-IEIVA 207
Sc.Hxk2  157 FSFPASQNKINEGILQRWTKGFDIPNIENHDV-VPMLQKQITKR-------NIP-IEVVA 207
Sc.Emi2  164 FSYPVDQTSLSSGTLIRWTKSFKIEDTVGKDV-VRLYQEQLDIQ-------GLSMINVVA 215
Sc.Glk1  163 FSYPVDQTSLNSGTLIRWTKGFRIADTVGKDV-VQLYQEQLSAQ-------GMPMIKVVA 214
                ▼
Sc.Unch  193 VINDAIAVSLTSKFICE-----------NDSISLIIGTGTNACFEVPYGYLPPFKRDALR 241
Sc.Hxk1  208 LINDTVGTLVASYYTDP-----------ETKMGVIFGTGVNGAFYDVVSDIEKLEG-KLA 255
Sc.Hxk2  208 LINDTTGTLVASYYTDP-----------ETKMGVIFGTGVNGAYYDVCSDIEKLQG-KLS 255
Sc.Emi2  216 LTNDTVGTFLSHCYTSGSRP-SSAGEISEPVIGCIFGTGTNGCYMEDIENIKKLPD-ELR 273
Sc.Glk1  215 LTNDTVGTYLSHCYTSDNTDSMTSGEISEPVIGCIFGTGTNGCYMEEINKITKLPQ-ELR 273

Sc.Unch  242 ETLPSSYNKETLNFKHVLINSEIGFIGKNVIALQP--FDIHGAI-----SYEMPLECVTS 294
Sc.Hxk1  256 DDIP--------SNSPMAINCEYGSFDNEHLVLPRTKYDVAVDEQ-SPRPGQQAFEKMTS 306
Sc.Hxk2  256 DDIP--------PSAPMAINCEYGSFDNEHV-VPRTKYDITIDEE-SPRPGQQTFEKMSS 305
Sc.Emi2  274 TRLLHE------GKTQMCINIEWGSFDNELKHLSATKYDIDIDQKFSPNPGYHLFEKRIS 327
Sc.Glk1  274 DKLIKE------GKTHMIINVEWGSFDNELKHLPTTKYDVVIDQKLSTNPGFHLFEKRVS 327

Sc.Unch  295 GKWLPLSLKNILLQYNI-------------IPKNF--PVEFNGELVCQLAEDCTNAWFE- 338
Sc.Hxk1  307 GYYLGELLRLVLLELNEKGLMLKD-----QDLTKLKQPYIMDTSYPARIEDDPFENLEDT 361
Sc.Hxk2  306 GYYLGEILRLALMDMYKQGFIFKN-----QDLSKFDKPFVMDTSYPARIEEDPFENLEDT 360
Sc.Emi2  328 GMYLGELLRNILVDLHARGLILGQYRNYDQLPHRLKTPFQLCSEVLSRIEIDDSTNLRET 387
Sc.Glk1  328 GMFLGEVLRNILVDLHSQGLLLQQYRSKEQLPRHLTTPFQLSSEVLSHIEIDDSTGLRET 387

Sc.Unch  339 -------------NEHYALICQIARLLIKRAAFYVAAIVQAIDIITG---CKNYNFIHIG 382
Sc.Hxk1  362 DDIFQKDFGVKTTLPERKLIRRLCELIGTRAARLAVCGIAAICQKR------GYKTGHIA 415
Sc.Hxk2  361 DDLFQNEFGINTTVQERKLIRRLSELIGARAARLSVCGIAAICQKR------GYKTGHIA 414
Sc.Emi2  388 ELSFLQSLRLPTTFEERKAIQNLVRSITRRSAYLAAVPIAAILIKTNALNKRYHGEVEIG 447
Sc.Glk1  388 ELSLLQSLRLPTTPTERVQIQKLVRAISRRSAYLAAVPLAAILIKTNALNKRYHGEVEIG 447

Sc.Unch  383 YVGSFLHNSNFYREQIKYY------------SSIHIKLQFLNHSNLLGAAIATYLNKSDN 430
Sc.Hxk1  416 ADGSVYNKYPGFKEAAAKGLRDIYGWTGDAS-NDPITIVPAEDGSGAGAAVIAALSEKRI 474
Sc.Hxk2  415 ADGSVYNRYPGFKEKAANALKDIYGWTQTSLDDYPIKIVPAEDGSGAGAAVIAALAQKRI 474
Sc.Emi2  448 FDGYVIEYYPGFRSMLRHALALSP---IGTEGERKIHLRLAKDGSGVGAALCALVA---- 500
Sc.Glk1  448 CDGSVVEYYPGFRSMLRHALALSP---LGAEGERKVHLKIAKDGSGVGAALCALVA---- 500

Sc.Unch  431 QVQ-------- 433
Sc.Hxk1  475 AEGKSLGIIGA 485
Sc.Hxk2  475 AEGKSVGIIGA 485
Sc.Emi2      -----------
Sc.Glk1      -----------
```

Fig. S1. **Comparison of amino acid sequences of YLR446Wp with the known hexokinases of *S. cerevisiae*.** Sc.Unch, YLR446Wp (AAT92658.1) and four hexokinases of *S. cerevisiae*: Sc.Hxk1 (NP_116711.3), Sc.Hxk2 (NP_011261.1), Sc.Glk1 (NP_009890.1) and Sc.Emi2 (NP_010804.3). ▼, putative catalytic residue; ▽, putative ATP binding residue. Multiple alignments of amino acid sequences were carried out using CLUSTAL O(1.2.4)[23].

Supplementary Fig.S2

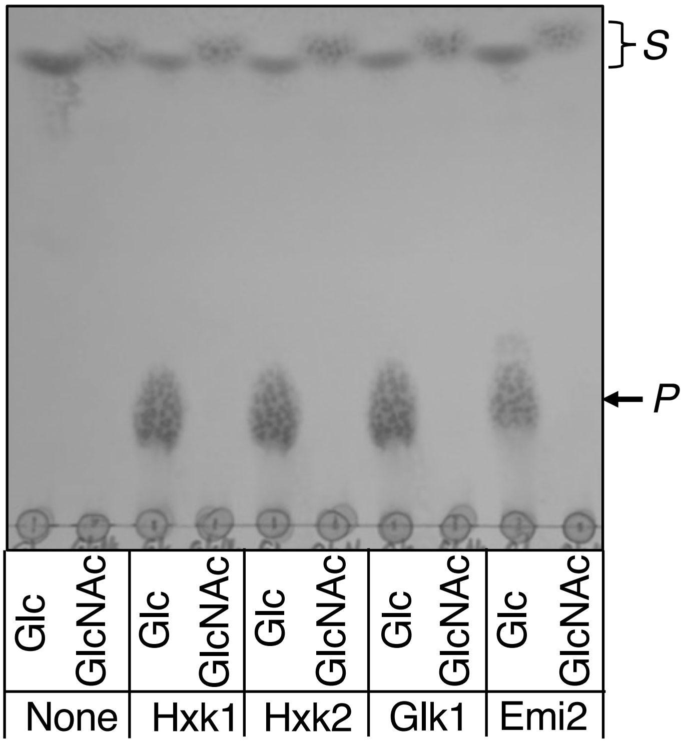

Fig. S2**. TLC analysis of GlcNAc and Glc phosphorylation activities of Hxk1, Hxk2, Glk1, and Emi2**. The reaction comprising either Glc or GlcNAc (10 mM each), 10 mM ATP, and 10 mM $MgCl_2$ with each recombinant enzyme was carried out for 30 min in 50 mM Tris-HCl (pH 8.0). None, the reaction without enzyme; *S*, sugar substrates; *P*, products.

Supplementary Fig.S3

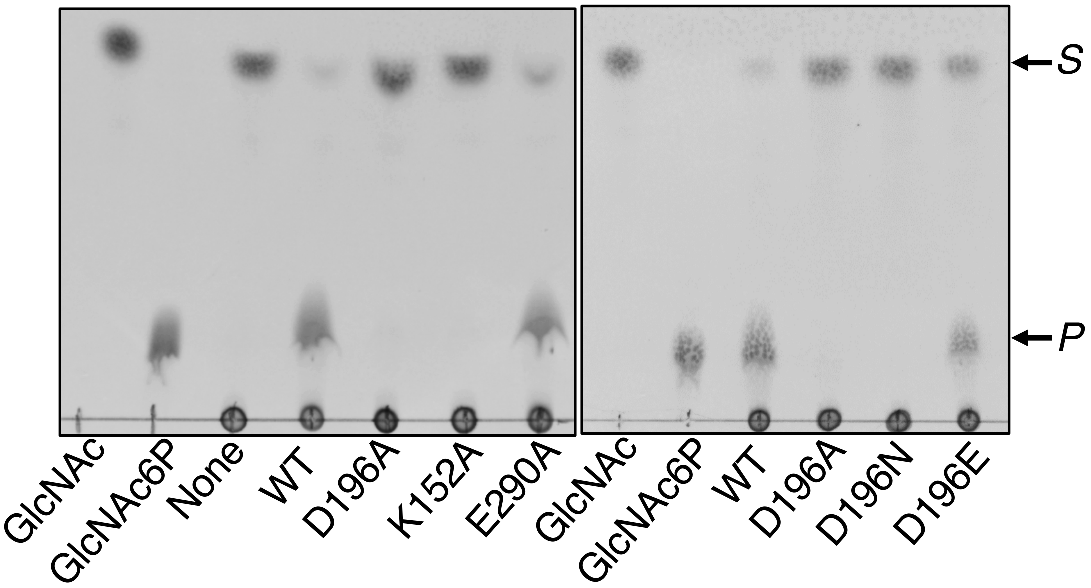

Fig. S3. **Effects of the mutations at putative catalytic and ATP-binding residues for Ngk1.** TLC analyses of GlcNAc kinase activity of Ngk1 and the mutant enzymes. A reaction comprising 10 mM GlcNAc, 10 mM ATP, and 10 mM $MgCl_2$ with each enzyme (0.1 mg/mL) was performed for overnight. WT, the wild-type Ngk1; None, no enzyme; K152A, D196A, D196N, D196E, and E290A are the Ngk1 mutant enzymes.
